# CYTOR drives prostate cancer progression via facilitating AR‐V7 generation and its oncogenic signalling

**DOI:** 10.1002/ctm2.1230

**Published:** 2023-05-02

**Authors:** Jianpeng Yu, Tianyu Shen, Yang Li, Tangxi Hao, Long Yang, Hanlin Li, Xuan‐Mei Piao, Zheng Zhang, Shimiao Zhu, Changyi Quan, Wun‐Jae Kim, Yang Zhao, Yuanjie Niu, Zhiqun Shang

**Affiliations:** ^1^ Tianjin Institute of Urology The Second Hospital of Tianjin Medical University Tianjin China; ^2^ School of Medical Nankai University Tianjin China; ^3^ Department of Urology Tianjin Medical University General Hospital Tianjin China; ^4^ Department of Urology, College of Medicine Chungbuk National University Cheongju Chungbuk South Korea

To the Editor:

There are no data regarding expressed and functional characterisation of cytoskeleton‐related non‐coding RNA has been reported in prostate cancer (PCa). Here, we report a cytoskeleton regulator RNA (CYTOR)‐regulated process that mediates castration‐resistant PCa (CRPC)‐specific androgen receptor splice variant 7 (AR‐V7) generation, and further explore the vulnerability of CRPC growth through CYTOR‐targeted locked nucleic acid (LNA).

We retrieved public castration‐sensitive PCa (CSPC) datasets (*n* = 65), neuroendocrine PCa (NEPC) datasets (*n* = 49) and CRPC datasets (including two studies, *n* = 171 and *n* = 118).^1^ Across the above RNA‐seq data, CYTOR was found to be upregulated in CRPC with low expression in CSPC and NEPC (Figure 1A). RNA in situ hybridisation (RISH) assays^2^ of our centre samples confirmed the public domain data (Figure 1B and Figure S1A). Consistent with tissue detection, androgen‐influenced CYTOR revealed significant increase in our two continuous established castration‐resistant cell lines: LNCaP‐AI,^3^ C4‐2 Enz‐R (Figure 1C and Figure S1B–G). Additionally, progression was more common in CSPC with higher CYTOR expression (Figure 1D). Expression analysis of CYTOR in flash‐frozen surgical specimens was conducted in 11 CRPC patients (Figure 1E). Patients with high expression of CYTOR received worse PSA response to subsequential enzalutamide than those with CYTOR low expression (Figure 1F). Then, gene functional assays suggested knockdown of CYTOR suppressed the cancer cells growth (Figure 1G–J). The above results hint the association of CYTOR with CRPC development and inferior clinical outcomes.

**FIGURE 1:**
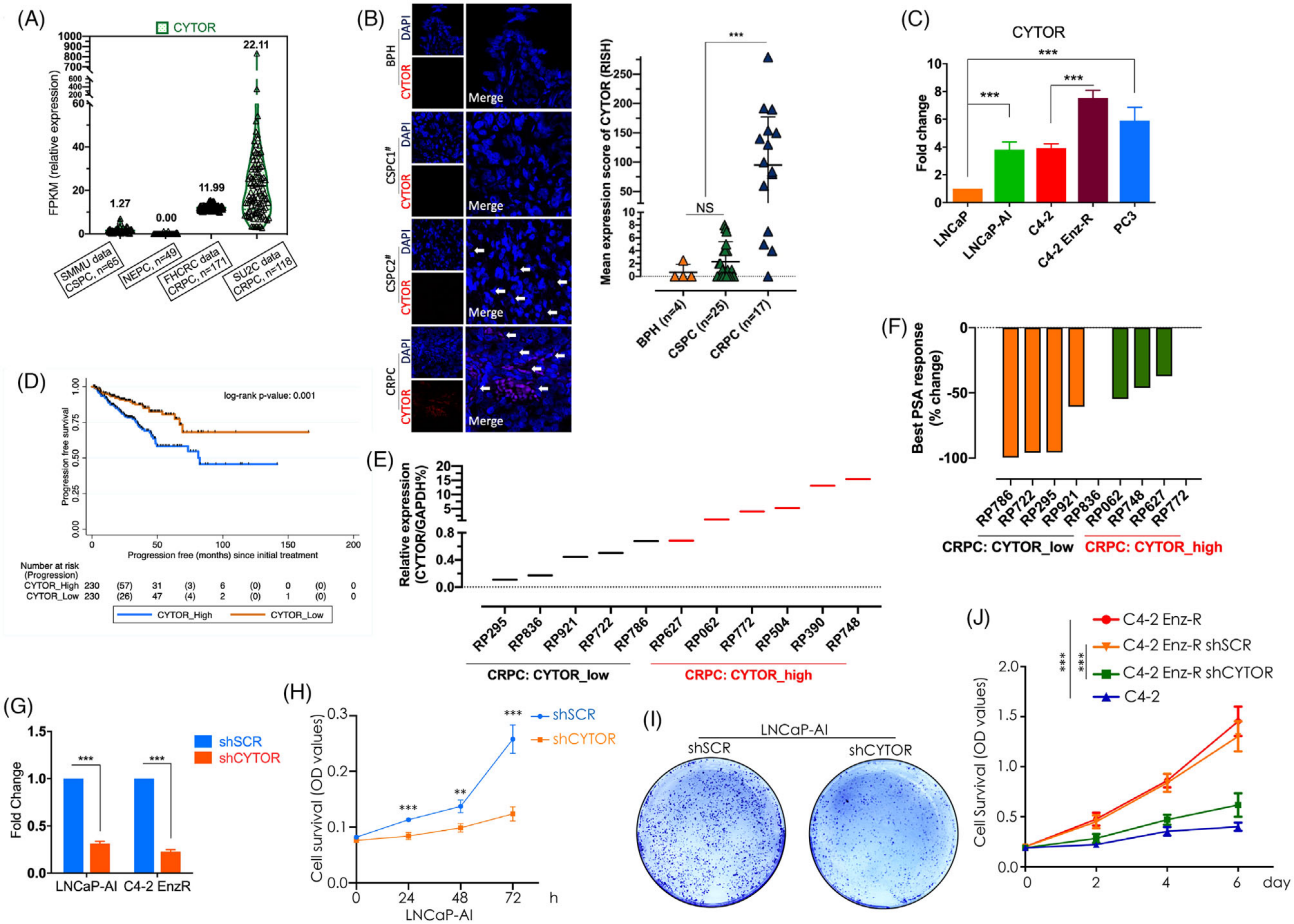
Increased expression of CYTOR is correlated with prostate cancer progression and development of castration resistance. (A) Relative expression (FPKM) of CYTOR across the indicated different cohorts. Boxplot definitions: the centre line depicts the median; the median values are on the top of the boxes. The boxes present CYTOR levels in castration‐sensitive prostate cancer (CSPC) tissues (SMMU team paper data [*n* = 65]), neuroendocrine prostate cancer (NEPC) tissues (*n* = 49) and castration‐resistant prostate cancer (CRPC) tissues (including FHCRC team paper data [*n* = 171] and SU2C/PCF Dream Team paper data [*n* = 118]). All the data is retrieved from cBioPortal database. FPKM: fragments per kilobase of transcript per million mapped reads. (B) RISH analysis of CYTOR expression in benign prostatic glands (benign, *n* = 4), CSPC (*n* = 25) and CRPC (*n* = 17) paraffin‐embedded tissues. Representative pseudo‐coloured images of CYTOR (red) are shown. Nucleus was stained with DAPI (blue). The barplot represents mean CYTOR expression scores (from RISH analysis), with vertical bars indicating the standard deviation of the means. NS: not significant. RISH: RNA in situ hybridisation. (C) qRT‐PCR detection of CYTOR expression in LNCaP, LNCaP‐AI, C4‐2, C4‐2 Enz‐R and PC3 cell lines, normalised by the level of GAPDH. LNCaP‐AI cell model was constructed following long‐term culture of the parental LNCaP cells under androgen‐deprived conditions until developed resistance to androgen deprivation. C4‐2 Enz‐R cell line was generated by culturing C4‐2 cells under increasing enzalutamide concentrations from 10 to 40 μM until developed resistance. A *p*‐value of less than .05 was considered significant. ***p < .001. (D) Kaplan–Meier curve of the progression‐free survival rates in castration‐sensitive PCa CYOTR high‐expressed patients and low‐expressed patients using RNA‐seq (from TCGA) median expression as cutoff. The Cancer Genome Atlas (TCGA) data were retrieved from cBioPortal web server. (E) Detection of CYTOR expression in 11 frozen CRPC samples via qRT‐PCR assays. These fresh‐frozen specimens were obtained from Chungbuk National University Hospital. All tumours were macro‐dissected, typically within 15 minutes of surgical resection. The expressions of CYTOR were normalised by the levels of GAPDH mRNA. Last six CRPC patients were put into CYTOR‐high expressed group (red fonts). (F) The best PSA responses (% drop of PSA from baseline following enzalutamide treatment) of the nine CRPC patients indicated in E. The four CYTOR highly expressed CRPC patients (RP062, RP748, RP627 and RP772) are presented with red fonts. Because patients RP504 and RP390, referred in the CYTOR_high group in E, refused to use continuous enzalutamide, the subsequential PSA response was not conducted in F. Patients RP836 and RP772 received no PSA response to enzalutamide, whose best PSA response was 0% change. PSA: prostate‐specific antigen. (G). qRT‐PCR detection of CYTOR in stable CYTOR‐knockdown LNCaP‐AI and C4‐2 EnzR cells. ***p < .001. (H) CYTOR stably knocked down LNCaP‐AI cells growth was assessed daily for 3 days using an MTT assay. **p < .01, ***p < .001; h: hour. (I) The ability of LNCaP‐AI cells to form colonies was determined by colony assays after CYTOR knockdown in the absence of androgen. (J) CYTOR stably knocked down C4‐2 Enz‐R cells display enhanced vulnerability when compared to the control cells. Cell growth was determined by MTT assays every 2 days. Cell survival was presented by optical density (OD, 490 nm) values. ***p < .001.

As it is, the primary therapeutic intervention for advanced PCa is androgen‐deprivation therapy (ADT) with the goal of castration to suppress androgen receptor (AR) signalling. Although most patients respond to ADT, some inevitably develop resistance and progress to CRPC because of AR‐V7 expression.^4^ Extensively investigated AR‐V7 is a typically truncated AR without the ligand‐binding domain but retaining transcriptional‐regulated activity to mediate ligand‐independent AR signalling.^5^ RNA‐seq analysis revealed many AR‐V7 downstream genes were differentially regulated as CYTOR knockdown (Figure 2A, Table S2). Most of them were enriched in metabolic pathways (Figure 2B). We validated the downregulation of AR‐V7 canonic‐activated genes (Figure 2C) after silencing CYTOR. Interestingly, knockdown of CYTOR resulted in specific decrease of AR‐V7 without concurrent decrease of full‐length AR (AR‐FL) (Figure 2D), suggesting the critical role of CYTOR in AR‐V7 mRNA splicing process. Multiplexed RISH assays of CRPC specimens revealed colocalisation and positive correlation of CYTOR and AR‐V7 (pre‐mRNA accumulated in nuclei) (Figure 2E). Their positive correlation was also confirmed by RT‐PCR in four flash‐frozen specimens (Figure 2F).

**FIGURE 2:**
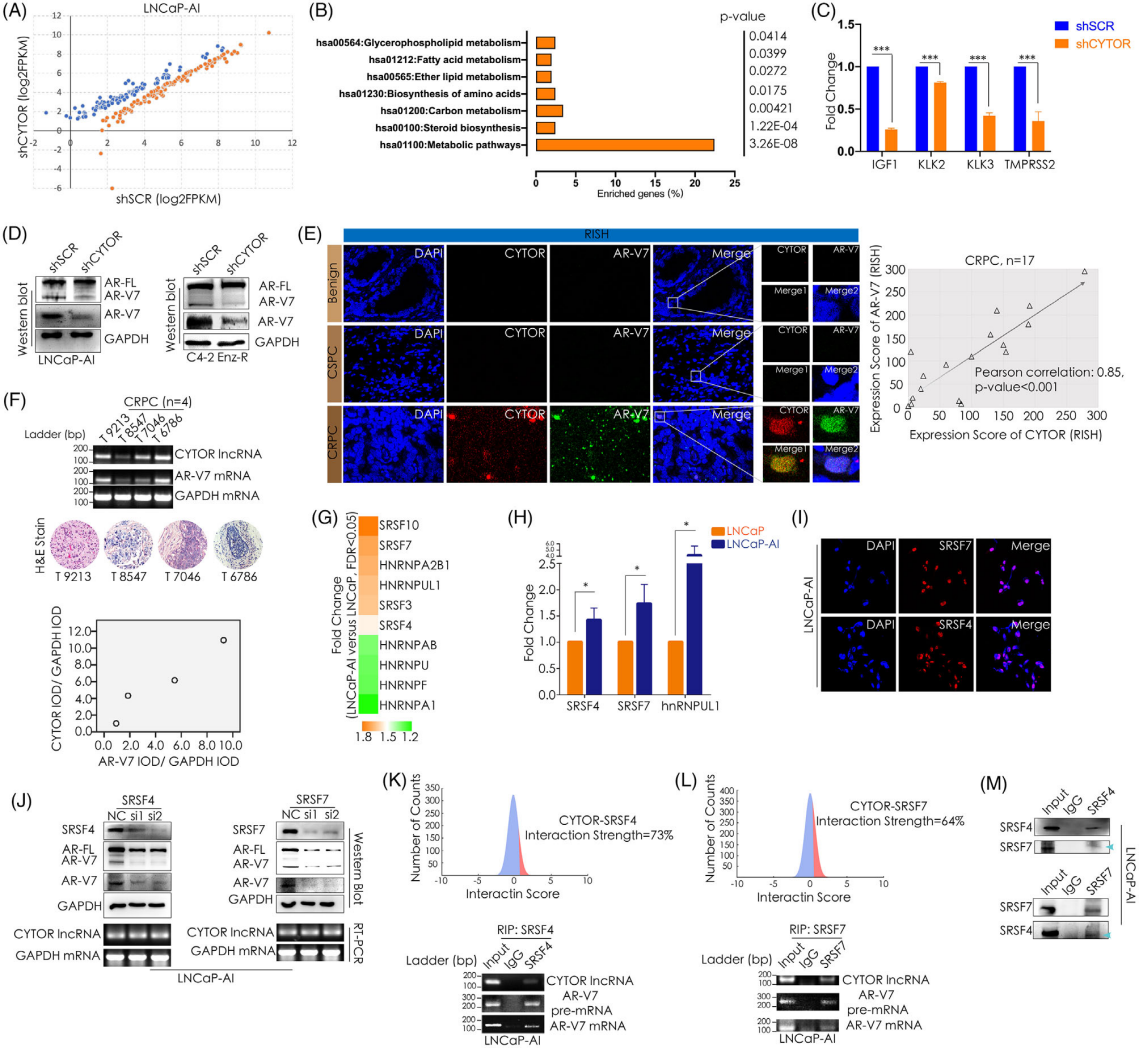
CYTOR interacts with SRSF4/7 proteins that promote AR‐V7 mRNA generation and its oncogenic signalling. (A) Scatter plots of AR‐V7 downstream genes upregulated (orange) or downregulated (blue) significantly (*p*‐value < .05) by CYTOR in LNCaP‐AI cells. X and Y axes are normalised signal values (log2 scaled) for each gene, according to the RNA‐seq data of shCYTOR versus shSCR in LNCaP‐AI cell line. SCR: scramble. AR‐V7 downstream genes were collected from Basil et al. study.^8^ (B) KEGG pathway enrichment of the genes presented in A. (C) qRT‐PCR validation of several differentially expressed AR‐V7 canonic downstream genes in A, normalised by the level of GAPDH. A *p*‐value of less than .05 was considered significant. ***p < .001. (D) Reduced expression of AR‐V7 after CYTOR stable knockdown. Western blot detection of AR‐FL and AR‐V7 in the indicated cell lines. AR‐FL: full‐length androgen receptor. (E) Multiplexed RISH analysis of CYTOR and AR‐V7 levels in benign prostatic glands (benign, *n* = 4), CSPC (*n* = 25) and CRPC (*n* = 17) paraffin‐embedded tissues. Representative pseudo‐coloured images of CYTOR (red) and AR‐V7 mRNA (green) are shown. Nucleus is stained with DAPI (blue). The nucleus green signals presented AR‐V7 pre‐mRNA. Association of CYTOR and AR‐V7 expression in CRPC (*n* = 17) tissues was analysed by Pearson correlation coefficient (Pearson correlation: .85, *p*‐value < .001) by SPSS software. (F) Detection of CYTOR and AR‐V7 mRNA expression in four fresh CRPC samples via RT‐PCR assays (upper). H&E stain images of the four CRPC samples to present the cancer tissues. GAPDH was used as a loading control. The four samples were collected from our hospital (the Second Hospital of Tianjin Medical University). Expression of GAPDH mRNA, CYTOR and AR‐V7 mRNA determined by ImageJ with integrated optical density (IOD) based on the results of RT‐PCR. Data normalised by GAPDH expression. (G) Heatmap of expression level fold changes for genes encoding SR proteins and hnRNPs upregulated in LNCaP‐AI cells when compared to the parental LNCaP cells by RNA microarray analysis. Details shown in our previous published study GSE124291. (H) qRT‐PCR detection of the differentially upregulated SR proteins and hnRNPs expression in LNCaP and LNCaP‐AI cell lines, normalised by the level of GAPDH. Data were obtained from three independent experiments. A *p*‐value of less than .05 was considered significant. *p < .05. (I) Confocal microscopy immunofluorescent images of SRSF4 and SRSF7 proteins in LNCaP‐AI cells. (J) Western blot analysis of SRSF4, SRSF7, AR‐FL, AR‐V7 and GAPDH expression, and RT‐PCR detection of CYTOR in LNCaP‐AI cells transfected with SRSF4 or SRSF7 siRNAs, respectively. NC: negative control. (K and L) Upper: the interaction strength of CYTOR–SRSF4 or CYTOR–SRSF7 pairs computed by the *cat*RAPID strength algorithm. Interaction strength values above 50% indicate high specificity for the interaction. The blue areas represent the interaction strength. Lower: SRSF4‐RIP or SRSF7‐RIP followed by RT‐PCR analysis to detect the interaction of SRSF4 or SRSF7 with CYTOR, AR‐V7 pre‐mRNA and AR‐V7 mRNA in LNCaP‐AI cells. IgG‐RIP assays were used as negative controls. RIP: RNA immunoprecipitation. (M) SRSF4‐IP or SRSF7‐IP followed by Western blot to determine the interaction between SRSF4 and SRSF7 proteins in LNCaP‐AI cells. IgG‐IP assays were used as negative controls. IP: immunoprecipitation.

Because key RNA‐binding protein families involved in alternative splicing may include serine/arginine‐rich proteins (SR proteins) and heterogeneous nuclear ribonucleoproteins (hnRNPs), we conducted differential expression analysis of SR proteins and hnRNPs between LNCaP‐AI and LNCaP cells by our published RNA‐arrays (GSE124291), and screened six upregulated splicing factors in LNCaP‐AI cells (>1.5‐fold) (Figure 2G). We further confirmed the upregulation of three genes (Figure 2H and Figure S2A). By Human Splicing Finder,^6^ the similar consensus splice site value for splice junctions of intron 3/cryptic exon 3 (CE3) (80.38) (as in AR‐V7) and intron 3/exon 4 (80.1) (as in AR‐FL) (Figure S2B) suggested the existence of a mechanism for CRPC‐specific CE3 splice site utilisation. Given the established role of SR proteins in binding to pre‐mRNA that prevents exon skipping, and the classical role of hnRNPs as splicing repressors, we postulated that nuclear‐localised SRSF4 and SRSF7 (Figure 2I) may repress CE3 skipping, thus ensuring the correct 5′ to 3′ linear order of exons (exon1‐3/CE3) in AR‐V7 mRNA. Indeed, knockdown of SRSF4 or SRSF7 resulted in decreased expression of AR‐FL and AR‐V7, while withoutimpact on CYTOR (Figure 2J). The catRAPID strength algorithm computed output suggested the high specificity of CYTOR–SRSF4 interaction and CYTOR–SRSF7 interaction, respectively (Figure 2K,L).^7^ RNA immunoprecipitation results revealed both SRSF4 and SRSF7 proteins interacted with CYTOR, AR‐V7 pre‐mRNA and AR‐V7 mRNA (Figure 2K,L), indicating nuclear binding of SRSF4 and SRSF7 to AR‐V7 pre‐mRNA and CYTOR was responsible for AR‐V7 generation, even though there was weak interaction of SRSF4 and SRSF7 (Figure 2M).

According to the functional interaction of CYTOR and SRSF4/7 proteins, we hypothesised that CYTOR may recognise AR‐V7 pre‐mRNA to induce this splicing process. Toward this end, maximum entropy modeling was used to collect motifs in the intron3/CE3 flanking sequence and identified the 3′ motif (3′ site of intron 3) and the 5′ motif (first 20 bp of CE3) (Figure 3A–C).^6^ The complementary sequence of the 5′ motif in the sequence of CYTOR (5′‐UUCCAACCGC‐3′) suggested that CYTOR may recognise the 5′ motif of CE3 (5′‐GGGUUGGCAA‐3′) to initiate the splicing process (Figure 3C). Next, we designed an 18 bp antisense oligonucleotides (ASO) to the 5′ motif of CE3 (ASO^CE3^) to prevent the recognition. The ASO^CE3^ suppressed, in a concentration‐ and time‐dependent manner, the expression of AR‐V7 mRNA (Figure 3D). We then designed an 18 bp ASO^CYTOR^ to the complementary sequence of CE3 5′ motif in CYTOR. ASO^CYTOR^ inhibited expression of AR‐V7 mRNA without interfering CYTOR expression (Figure 3E and Figure S2C). Also as shown in C4‐2 Enz‐R cells, the ASO^CE3^ and ASO^CYTOR^ prevented the generation of AR‐V7 mRNA (Figure 3F,G). To validate this splicing model, truncated mutant assays confirmed the pivotal role of 5′‐UUCCAACCGC‐3′ in CYTOR on AR‐V7 expression (Figure S2D,E). Together, CYTOR/SRSF4/SRSF7 complex interacts with AR‐V7 pre‐mRNA to regulate its splicing by recognising a specific signal element in CE3 (Figure 3H).

**FIGURE 3:**
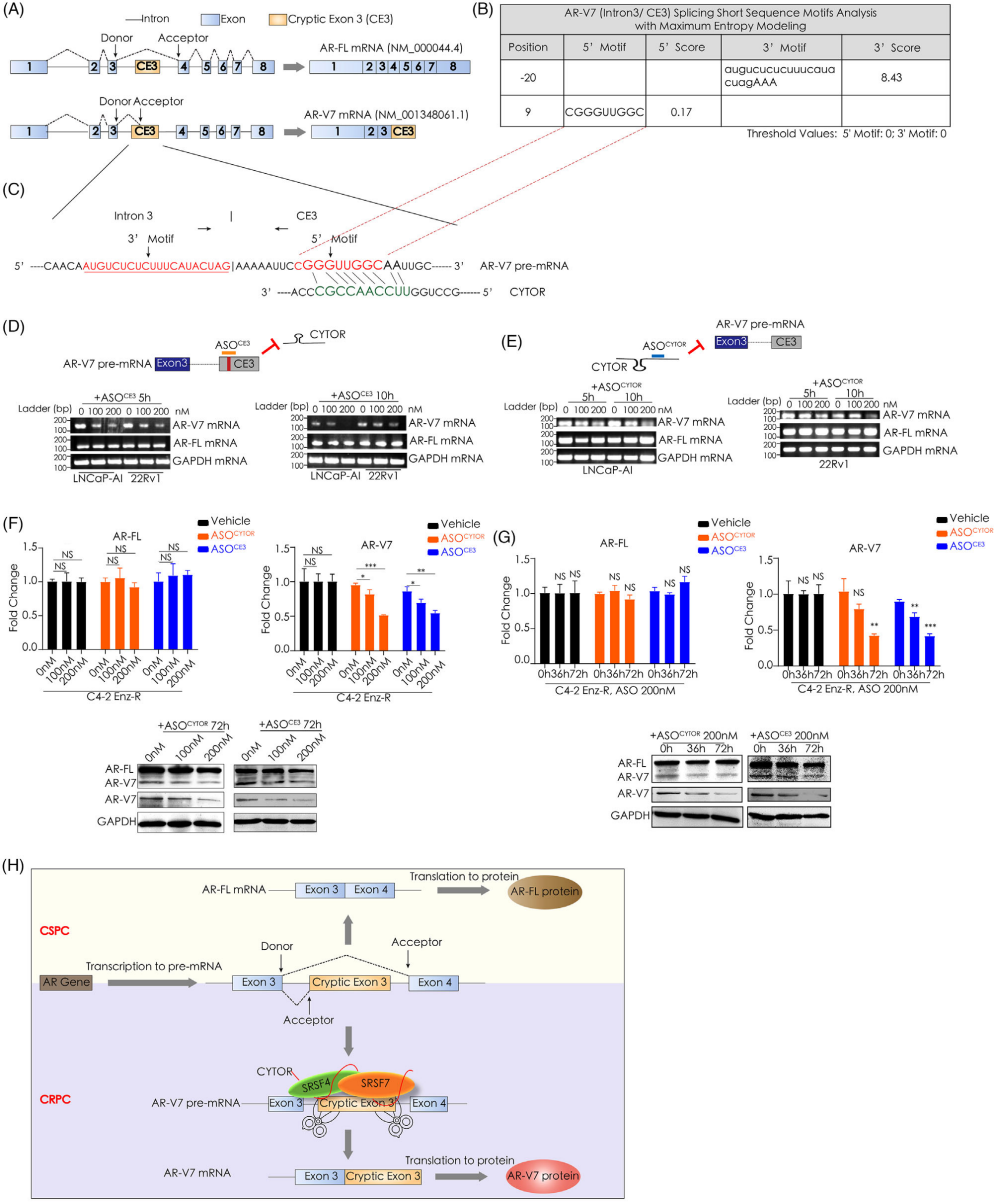
Recognition of AR‐V7 cryptic exon 3 by CYTOR is critical for AR‐V7 generation. (A) Transcript structure of AR‐FL and AR‐V7. Left: schematic representation of the exons and introns for AR‐FL and AR‐V7. CE3: cryptic exon 3. Right: retained exons in AR‐FL and AR‐V7 mRNA. AR‐FL, full‐length androgen receptor. (B and C) In silico prediction of AR‐V7 CE3 splicing motifs via Maximum Entropy Modeling in Human Splicing Finder tool (http://www.umd.be/HSF3/). The underlined nucleotides coloured in red represent the 3′ motif and 5′ motif in the intron3/CE3 splicing site. Lower case in table: intronic sequences; Upper case in table: exonic sequences; CE3: cryptic exon 3. 3′ motif represents 3′ site of intron3 in intron3/CE3 boundary; 5′ motif represents the 5′ site of CE3. (D) Inhibition of AR‐V7 generation in LNCaP‐AI and 22Rv1 cells using an antisense oligonucleotide (ASO^CE3^) targeting the predicted 5′ motif in CE3 presented in C. Schematic representation of ASO^CE3^ targeting process (upper). RT‐PCR analysis of AR‐V7 mRNA and AR‐FL mRNA levels in these two cell lines transfected with indicated concentration of ASO^CE3^ (lower). GAPDH was used as a loading control. CE3: cryptic exon 3; h: hour. (E) Inhibition of AR‐V7 generation in LNCaP‐AI and 22Rv1 cells using an antisense oligonucleotide (ASO^CYTOR^) targeting the complementary sequence of the predicted CE3 5′ motif in CYTOR. Schematic representation of ASO^CYTOR^ targeting process (upper). RT‐PCR analysis of AR‐V7 mRNA and AR‐FL mRNA levels in these two cell lines transfected with indicated concentration ASO^CYTOR^ (lower). GAPDH was used as a loading control. CE3: cryptic exon 3; h: hour. (F and G) Inhibition of AR‐V7 expression in C4‐2 Enz‐R cells using the ASO^CE3^ or ASO^CYTOR^. qRT‐PCR analysis (upper) and Western blot detection (lower) of AR‐V7 and AR‐FL levels in these cells transfected with indicated concentration of ASOs for different times. GAPDH was used as a loading control. CE3: cryptic exon 3; h: hour; NS: not significant. *p < .05, **p < .01, ***p < .001. (H) A model for assumed CYTOR‐mediated splicing of AR‐V7 mRNA in CRPC. Additionally, AR‐FL mRNA generation process in CSPC was presented. CRPC: castration‐resistant prostate cancer; CSPC: castration‐sensitive prostate cancer; AR‐FL: full‐length androgen receptor.

Then LNAs GapmeR^CYTOR^ were designed to silence CYTOR (Figure 4A). In C4‐2 Enz‐R cells, AR‐V7 expression was largely suppressed in parallel with the silenced pattern of CYTOR in a concentration‐ and time‐dependent manner (Figure 4B–E). GapmeR^CYTOR^ could attenuate the resistance of enzalutamide significantly in vitro (Figure 4F). We then established in vivo mouse models and found that enzalutamide significantly suppressed C4‐2 tumours, shCYTOR‐ and GapmeR^CYTOR^‐treated C4‐2 Enz‐R tumours (Figure 4G,H). The expressions of CYTOR and AR‐V7 were validated in each group (Figure S2F,G). As such, our data suggested that on‐target effect of CYTOR knockdown with GapmeR^CYTOR^ can be used as an option in castration resistance to provide therapeutic efficacy.

**FIGURE 4:**
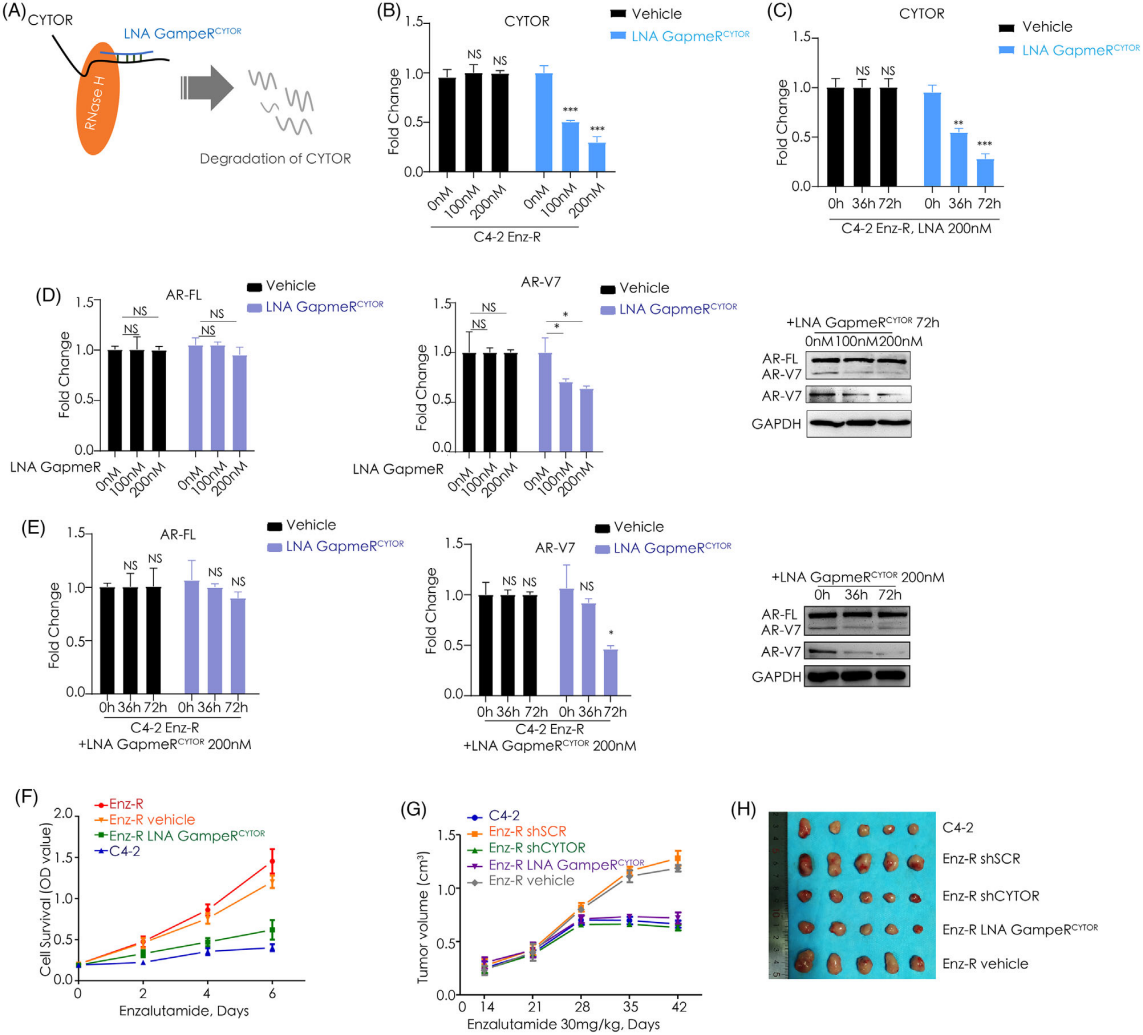
Effects of targeting CYTOR on CRPC cells in vitro and in vivo growth using shRNA or locked nucleic acid (LNA). (A) Silence of CYTOR by LNA GapmeR^CYTOR^ targeting the same sequence as ASO^CYTOR^. Schematic representation of LNA GapmeR^CYTOR^ targeting process. LNA: locked nucleic acid. (B and C) Reduced expression of CYTOR after CYTOR knocked down by LNA GapmeR^CYTOR^. qRT‐PCR analysis of CYTOR expressed levels in C4‐2 Enz‐R cells transfected with indicated concentration LNA GapmeR^CYTOR^ for different times. GAPDH was used as a loading control. h: hour; NS: not significant; LNA: locked nucleic acid. **p < .01, ***p < .001. (D and E) Reduced expression of AR‐V7 after CYTOR knocked down by LNA GapmeR^CYTOR^. qRT‐PCR analysis and Western blot detection of AR‐FL and AR‐V7 expression levels in C4‐2 Enz‐R cells transfected with indicated concentration LNA GapmeR^CYTOR^ for different times. GAPDH was used as a loading control. h: hour; NS: not significant; LNA: locked nucleic acid. *p < .05. (F) CYTOR knocked down C4‐2 Enz‐R cells display enhanced vulnerability when compared with the control cells. C4‐2 Enz‐R cells were transfected by vehicle or LNA GapmeR^CYTOR^, respectively. Cell growth was determined by MTT assays every 2 days. Cell survival is presented by optical density (OD, 490 nm) value. The cells were cultured with enzalutamide to target androgen receptor. (G) Subcutaneous tumour growth assays were performed with C4‐2 parental cells, C4‐2 Enz‐R cells, C4‐2 Enz‐R shSCR cells and C4‐2 Enz‐R shCYTOR cells. After 4 weeks, 25 injected mice developed tumours, five randomly chosen C4‐2 Enz‐R tumours were injected with vehicle LNA and another five randomly chosen C4‐2 Enz‐R tumours were injected with LNA GapmeR^CYTOR^ (10 mg/kg) in the inoculated site every day for 12 days. All mice were intraperitoneally injected with enzalutamide during the therapies. LNA: locked nucleic acid; shSCR: scramble shRNA. (H) Image of mice tumours from subcutaneously xenografting equal numbers of prostate cancer cells treated as indicated therapies.

In conclusion, we propose the importance of a novel complex composed of CYTOR/SRSF4/SRSF7 that mediates AR‐V7 generation, and a critical role in suppressing PCa progression by targeting CYTOR/AR‐V7 axis with shRNA or preclinical LNA Gapmer^CYTOR^.

## CONFLICT OF INTEREST STATEMENT

The authors declare they have no conflicts of interest.

## FUNDING INFORMATION

National Natural Science Foundation of China, Grant Numbers: 91959114, 81872106, 82072851, 81872100, 81972654, 82273262; National Natural Science Foundation of China, International (Regional) Cooperation and Exchange Program, Grant Number: 82061160493; Natural Science Foundation of Tianjin, Grant Numbers: 18PTLCSY00030, 21JCQNJC01700; Tianjin Key Medical Discipline (Specialty) Construction Project, Grant Numbers: TJYXZDXK‐023A, TJYXZDXK‐065B; The Second Hospital of Tianjin Medical University, Grant Number: 2020ydey01; Scientific Research Project of Tianjin Education Commission, Grant Number: 2021KJ225

## Supporting information

Supporting Information

Supporting Information

Supporting Information

Supporting Information

Supporting Information
