# Supplementary figures and images for: CYTOR drives prostate cancer progression via facilitating AR‐V7 generation and its oncogenic signalling
Source: Clin Transl Med. 2023 May 2;13(5):e1230. doi: 10.1002/ctm2.1230 (PMC10154880; doi:10.1002/ctm2.1230)

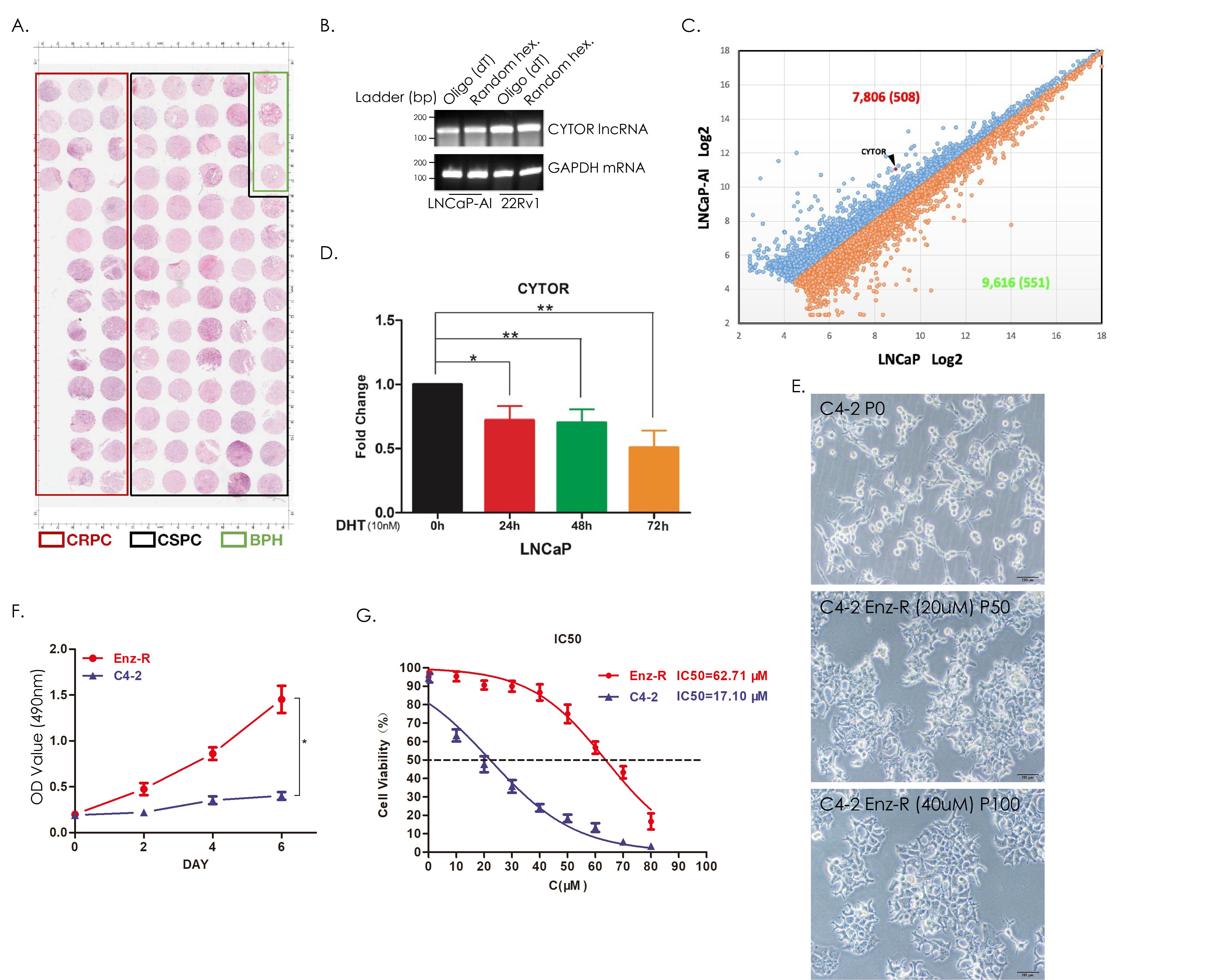

Supplement: Supplementary file 1 — Supporting Information [file CTM2-13-e1230-s002.jpg]

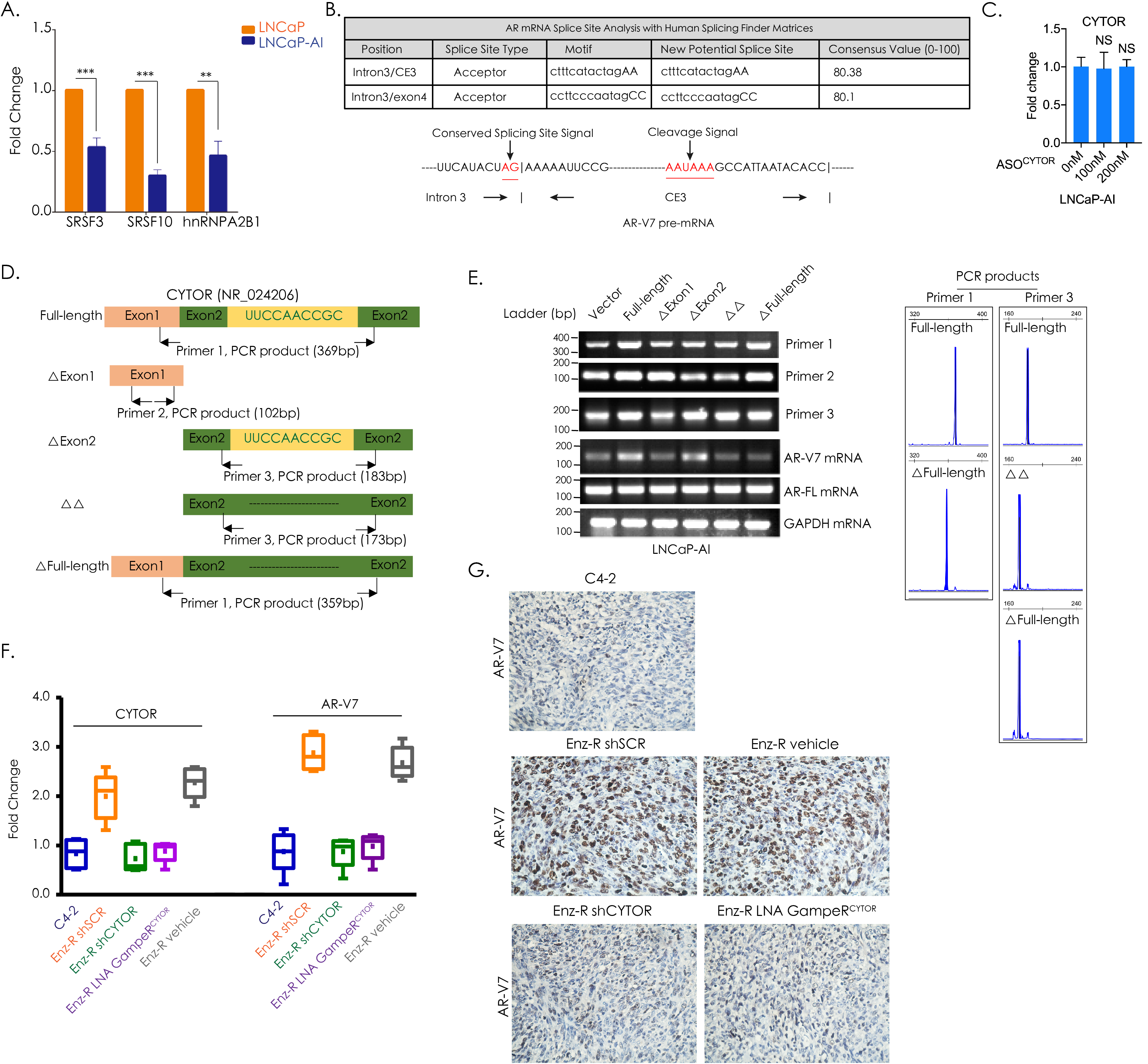

Supplement: Supplementary file 2 — Supporting Information [file CTM2-13-e1230-s004.jpg]
