# Supplementary material for: CYTOR drives prostate cancer progression via facilitating AR‐V7 generation and its oncogenic signalling
Source: Clin Transl Med. 2023 May 2;13(5):e1230. doi: 10.1002/ctm2.1230 (PMC10154880; doi:10.1002/ctm2.1230)
Supplement: Supplementary file 3 — Supporting Information [file CTM2-13-e1230-s003.docx]

**Methods：**

**Cell culture, antibodies, primers, and reagents.**

LNCaP and 22Rv1 cells were obtained from the American Type Culture Collection (ATCC, Manassas, VA, USA), and the LNCaP-AI cell model was constructed following long-term (more than one year) culture of the parental LNCaP cells under androgen deprived conditions. The LNCaP-AI cells were maintained in RPMI 1640 medium (Gibco, Waltham, MA, USA) supplemented with 10% charcoal-stripped fetal bovine serum (BI, Cromwell, CT, USA) [1, 2]. LNCaP and 22Rv1 cells were cultured in RPMI-1640 medium supplemented with 10% fetal bovine serum (Gibco), 100 ng/ml streptomycin and 100 U/ml penicillin (Gibco). Antibodies dilutions, company names and catalogue numbers are listed below. Anti-SRSF4 (A303-670A, 1:1000 dilution, Bethyl Laboratories Inc., Montgomery, TX, USA), anti-SRSF7 (A303-773A-M, 1:1000 dilution for western blot, Bethyl Laboratories, Inc.; ab137247, for IP and RIP assays, abcam, Cambridge, MA, USA), anti-AR-V7 (31-1109-00, 1:1000 dilution, RevMAb Biosciences, San Francisco, CA, USA), anti-Androgen Receptor (ab74272, 1:1000 dilution, abcam) and anti-GAPDH (sc-32233, 1:5000 dilution, Santa Cruz Biotechnology, Birmingham, AL, USA). The primers used in RT-PCR and qRT-PCR analysis were purchased from Sangon Biotech (Shanghai, China) and were listed in Supplementary Table 1.

**Generation of enzalutamide resistant cell lines.**

C4-2 enzalutamide resistant (Enz-R) cells were generated by culturing C4-2 cells under increasing enzalutamide concentrations from 10μM to 40μM. Specifically, C4-2 cells were cultured in 10uM enzalutamide for 35 passages, then the concentration was increased to 20uM. When the C4-2 cells were cultured with 20uM enzalutamide to passage 70, the concentration was increased to 40uM.

**Transfections.**

All cell transfections with siRNAs (purchased from GenePharma, Suzhou, China) were carried out using Lipofectamine (ThermoFisher Scientific, Waltham, MA, USA) with cells at 50% confluence cultured in 6-well plate. Plasmids were transfected via lentiviral vector purchased from GeneChem (Shanghai, China). The target sequences were listed in Supplementary Table 1.

**Cell growth assay.**

Cell growth was monitored by the 3-(4,5-dimethylthiazol-2-yl)-2,5-diphe-nyltetrazolium bromide proliferation assay. Each experiment was performed with cells seeded in 96-cell plate in triplicate.

**ASOs and LNAs design and usage.**

Antisense oligonucleotides (ASOs) were designed against the motif in AR-V7 or CYTOR to prevent the splicing motifs usage. The oligonucleotides were modified with 2’-O-methyl (sequences: ASO^CE3^ 5’-CAAUUGCCAACCCGGAAU-3’, ASO^CYTOR^ 5’-GUGGGCGGUUGGAACCAG-3’). LNA Gapmer^CYTOR^ and LNA vehicle were established with full phosphorothioate-modified backbone and 2’-O-methyl in two flanks 5 bases. All LNA Gapmers were purchased from Ribobio (Guangzhou, China) (sequences: LNA Gapmer^CYTOR^ mG*mU*mG*mG*mG*C*G*G*T*T*G*G*A*mA*mC*mC*mA*mG, LNA vehicle mC*mC*mU*mU*mC*C*C*T*G*A*A*G*G*T*T*mC*mC*mU*mC*mC, *= phosphorothioate backbone). Cells were transfected with the indicated amount of the oligonucleotides for indicated hours. For the reverse transcription step, the RNAs were pre-heated in the reaction mix (65 ℃/5min, 75 ℃/2min, 35 ℃/30s) prior to addition of the reverse transcriptase and RT reaction.

**Western blot analysis.**

Western blot analysis was performed by standard SDS-PAGE. Whole-cell lysates were prepared from 0.5-5 X 10^6^ cells in lysis buffer (25 mM Tris-HCl pH 7.4, 150 mM NaCl, 1 mM EDTA, 1% NP-40 and 5% glycerol). Lysates were clarified by centrifugation at 4 ℃ for 20 minutes in a Beckman tabletop centrifuge at 12000 rpm speed. Protein concentration of the lysates was determined by BCA Assay. Typically, 30-60ug of whole-cell lysates were separated on 10% acrylamide mini gels and transferred to Immobilon-P membrane (Millipore, Temecula, CA, USA). The membrane was blocked for 1 hour in 5% non-fat milk followed by an overnight incubation with primary antibody diluted in the same blocking buffer. After extensive washing, the membrane was incubated with secondary antibody for 1 hour, washed, and processed using the ECL Western blotting detection system (Tanon 4500).

**RNA isolation and cDNA synthesis.**

Total RNA from cell lines and fresh human tissues were isolated using TRIzol^TM^ Reagent (Invitrogen, Waltham, MA, USA) as standard RNA isolation procedures. The first strand cDNA synthesis was performed with the Reverse Transcription System (Roche, Indianapolis, IN, USA) following the manufacturer’s protocols.

**RT–PCR (reverse transcription PCR) and qRT-PCR (quantitative real-time PCR) analysis.**

Cell lines or tissues resulting cDNA was analyzed by RT-PCR or qRT-PCR using BIOER PCR system (Thermo Scientific) and PCR MasterMix (CWBIO, Beijing, China), or Applied Biosystems 7900 Real Time PCR System (Thermo Scientific) and SYBR Green PCR Master Mix (Roche) respectively, according to the manufacturers’ instructions. GAPDH was used as an internal control. The relative expression of RNAs was calculated using the comparative Ct method normalized by GAPDH level. Results of tissue PCR were valued by integrated optical density (IOD) with Image-J and normalized by IOD of GAPDH.

**Immunofluorescence, RISH and imaging.**

For immunofluorescence assays, LNCaP-AI cells were grown on cover glasses in 24-well plate and fixed with 4% paraformaldehyde in PBS for 15 minutes, after permeabilization with 0.2% Triton X-100 for 10 minutes and incubation with blocking buffer (PBS with 5%BSA) for 20 minutes, the cells were incubated with SRSF4 antibody or SRSF7 antibody overnight at 4 °C, and then with Alexa-Fluor-labeled 647 donkey anti-rabbit antibody (Life Technologies, Waltham, MA, USA) at room temperature for 2 hours. Cell nucleus was stained with DAPI. The stained cover glasses were mounted on standard slides and examined under Olympus FV1000D microscope.

RISH assays were performed on human tissue microarray sections with RNAscope® Multiplex Fluorescent Assay v2 (323110) from Advanced Cell Diagnostics. Briefly, tissue slides were boiled in pretreatment buffer for 30 minutes and rinsed in water. Next, AR-V7 mRNA and lncRNA-CYTOR target probes (purchased from Advanced Cell Diagnostics, Newark, CA, USA) were hybridized to AR-V7 mRNA and lncRNA-CYTOR at 40 °C for 2 hours, with these probes creating a binding site for a preamplifier. After this incubation, the preamplifier was hybridized to the target probes. FITC was assigned to AR-V7 probes and Cy3 was assigned to lncRNA-CYTOR probes. Cell nucleus was stained with DAPI. In total, 46 formalin-fixed, paraffin-embedded (FFPE) tissue samples were included (4 from benign prostatic hypertrophy, 25 from androgen castration-sensitive PCa and 17 from CRPC). AR-V7 mRNA and lncRNA-CYTOR signals were examined in morphologically intact cells and scored manually by a researcher with 400-fold magnification, using a reported expression value scoring system (18,19). Specific RISH signal was identified as punctate dots, and expression level was scored as follows: 0 = no staining or less than 1 dot per 10 cells, 1 =1 to 3 dots per cell, 2 =4 to 9 dots per cell (few or no dot clusters), 3 =10 to 14 dots per cell (less than 10% in dot clusters), and 4 = greater than 15 dots per cell (more than 10% in dot clusters). For each evaluable tissue core, a cumulative RISH product score was calculated as the sum of the individual products of the expression level (0 to 4) and percentage of cells (0 to 100) (i.e., [A%× 0] + [B%× 1] + [C%× 2] + [D% × 3] + [E% × 4]; total range =0 to 400). For each tissue sample, the RISH product score was averaged across evaluable tissue microarray cores.

**RNA-immunoprecipitation (RIP) and Immunoprecipitation (IP) assays.**

The RIP assays were performed according to manufacturer’s protocol of Magna RIP™ RNA-Binding Protein Immunoprecipitation Kit (Millipore). LNCaP-AI cells were seeded in 15cm plates, and one RIP reaction was performed using 10^8^ cells. Briefly, cells were harvested and lysed in RIP Lysis Buffer (10mM HEPES, 100mM KCl, 5mM MgCl2, 0.5% NP-40, 1mM DTT, 5mM PMSF, supplemented with protease inhibitors cocktail and RNase inhibitors) for 5 minutes at 4 °C and followed by centrifugation at 13,000 x g for 10 minutes. The cleared supernatant was collected, and protein concentration was determined by BCA Assay. Meanwhile, 50ul Magnetic Beads Protein A/G were coated by 5μg of SRSF4 or SRSF7 antibodies or control IgG at room temperature for 30 minutes with rotation. The coated Beads were washed and incubated with 100ul of the cell lysate, 35ul of 0.5M EDTA, 5ul of RNase inhibitor and 860ul of RIP Wash Buffer under moderate agitation overnight at 4°C. In the next day, wash the cell lysate/beads complex and re-suspend beads with 150 ul of proteinase K buffer containing 117 ul of RIP Wash Buffer, 15 ul of 10% SDS, 18 ul of 10 mg/mL proteinase K for 30 minutes at 55°C with shaking to digest the protein. After the incubation, transfer the supernatant into a new tube and add 400 ul of phenol: chloroform: isoamyl alcohol to each tube to perform RNA collected process and subjected to RT-PCR analysis.

LNCaP-AI cells were incubated with Pierce IP Lysis Buffer (Thermo Scientific) (25 mM Tris-HCl pH 7.4, 150 mM NaCl, 1 mM EDTA, 1% NP-40 and 5% glycerol) for 30 minutes at 4 °C and followed by centrifugation for 15 minutes at 12,000 rpm to perform Co-IP assays. Protein concentration of the lysates was determined by BCA Assay. For Each experiment, 50ul Magnetic Beads Protein A/G were coated by 5μg of SRSF4 or SRSF7 antibodies or control IgG at room temperature for 30 minutes with rotation. 500ug protein were combined with antibodies coated Beads, then incubated under moderate agitation overnight at 4°C. Beads were collected with the magnet, washed five times with lysis buffer and resuspended in lysis buffer. Following boiling with loading buffer (5×) (50% glycerol and bromophenol blue/xylene cyanole) for 5 minutes at 95 ℃, immunoprecipitants were subjected to SDS-PAGE and standard immunoblotting process.

**RNA-microarray processing and analysis.**

LNCaP-AI and LNCaP cell lines were used for this microarray analysis. RNA quantity and quality were measured by NanoDrop ND-1000 (Thermo Scientific). RNA integrity was assessed by standard denaturing agarose gel electrophoresis. Arraystar Human LncRNA Microarray V3.0 is designed for the global profiling of human lncRNAs and protein-coding transcripts, which is updated from the previous Microarray V2.0. About 30,586 lncRNAs and 26,109 coding transcripts were queried by this third generation LncRNA microarray. Sample labeling and array hybridization were performed according to the Agilent One-Color Microarray-Based Gene Expression Analysis protocol (Agilent Technology, Santa Clara, CA, USA) with minor modifications. Briefly, mRNA was purified from total RNA after removal of rRNA (mRNA-ONLY™ Eukaryotic mRNA Isolation Kit, Epicentre, Chicago, IL, USA). Then, each sample was amplified and transcribed into fluorescent cRNA along the entire length of the transcripts without 3' bias utilizing a random priming method (Arraystar Flash RNA Labeling Kit, Arraystar, Rockville, MD, USA). The labeled cRNAs were purified by RNeasy Mini Kit (Qiagen, Hilden, Sweden). The concentration and specific activity of the labeled cRNAs (pmol Cy3/μgcRNA) were measured by NanoDrop ND-1000. Each labeled cRNA (1 μg) was fragmented by adding 5 μl 10 × Blocking Agent and 1 μl of 25 × Fragmentation Buffer, heated at 60°C for 30 min, and finally mixed with 25 μl 2× GE Hybridization buffer. The hybridization solution (50 μl) was dispensed into the gasket slide and assembled to the LncRNA expression microarray slide. The slides were incubated for 17 hours at 65°C in an Agilent Hybridization Oven. Agilent Feature Extraction software (version 11.0.1.1) was used to analyze acquired array images. Quantile normalization and subsequent data processing were performed with using the GeneSpring GX v12.1 software package (Agilent Technologies). After quantile normalization of the raw data, lncRNAs that at least 3 out of 6 samples have flags in Present or Marginal (“All Targets Value”) were chosen for further data analysis. Differentially expressed lncRNAs with statistical significance between the two groups were identified through P-value/FDR filtering. Differentially expressed lncRNAs between the two samples were identified through Fold Change filtering. The microarray data was deposited in public database with accession ID GSE124291.

**Human samples.**

Prostate tissue specimens used in this study were surgical specimens from patients with prostate cancer with complete clinicopathological data. Castration-sensitive PCa specimens (n=25) were acquired by radical prostatectomy and CRPC specimens (n=17) were acquired by transurethral resection of the prostate. These samples were paraffin-embedded and subjected to RISH assays. In addition, 4 CRPC samples (collected from our hospital) acquired by transurethral resection of the prostate were fresh frozen in liquid nitrogen and processed for RT-PCR. Another 11 fresh-frozen specimens’ data were obtained from Chungbuk National University Hospital. These 11 tumors were macro-dissected, typically within 15 minutes of surgical resection. All studies were approved by the Ethics Committee of the Second Hospital of Tianjin Medical University, and informed consent was obtained from all patients. This study was also approved by the Institutional Review Board at Chungbuk National University (GR2010-12-010), and the experiments were undertaken with the informed written consents of all participants. The clinical data is available from the corresponding author upon reasonable request.

**In vivo experiments.**

The animal studies were approved by Tianjin Institute of Urology, Tianjin, China. Twenty-five male nude mice (6 weeks old) were purchased from Beijing HFK Bioscience Co. Ltd. (Beijing, China). Subcutaneous tumor growth assays were performed with C4-2, C4-2 Enz-R, C4-2 Enz-R shSCR and shCYTOR stable cell lines (5×10^6^ indicated cells injected to these mice separately). After four weeks, 25 injected mice developed tumors, 5 randomly chosen C4-2 Enz-R tumors were injected with vehicle LNA and another 5 randomly chosen C4-2 Enz-R tumors were injected with LNA GapmeR^CYTOR^ (10mg/kg) in the inoculated site every day for 12 days. All mice were intraperitoneal injected with enzalutamide during the therapies.

**Analysis of public datasets.**

The level (RNA-Seq data) of CYTOR was retrieved from 65 castration-sensitive PCa (CSPC) cases, 49 NEPC cases, 171 CRPC cases and 118 CRPC cases reported by the SMMU team paper [3], a Multi-institute paper [4], the FHCRC team paper [5] and SU2C/PCF Dream Team paper [6] respectively with FPKM (fragments per kilobase of transcript per million mapped reads) expression (from cBioPortal online-tool [7, 8]).

Progression free survival data of CSPC was collected from TCGA PCa database. All the patients were separated into CYTOR high-level group and low-level group with the median expression (RNA-seq data) as cutoff. Then the progression-free survival trends and curves were calculated by the Kaplan–Meier method and differences were evaluated using the log-rank test.

**Statistical analysis:**

Progression-free survival trends and curves were calculated by the Kaplan–Meier method and differences were evaluated using the log-rank test. Summary data were expressed as mean/median ± S.D. The student’s t and ANOVA tests were used to compare experimental groups. Pearson correlation coefficient analysis were performed by SPSS. A p value of <0.05 (two-sided) was considered to indicate a statistically significant difference. *, p<0.05; **, p<0.01; ***, p<0.001. All statistical analysis was performed with SPSS 22 statistical software (SPSS, IBM Corporation, Armonk, NY, USA) and StataSE 15.1 (StataCorp LLC, Texas, USA). FDR is calculated from Benjamin Hochberg FDR.

**References**

1. Shang Z, Yu J, Sun L et al. LncRNA PCAT1 activates AKT and NF-kappaB signaling in castration-resistant prostate cancer by regulating the PHLPP/FKBP51/IKKalpha complex. Nucleic Acids Res 2019; 47: 4211-4225.

2. Yu J, Sun L, Hao T et al. Restoration of FKBP51 protein promotes the progression of castration resistant prostate cancer. Ann Transl Med 2019; 7: 729.

3. Ren S, Wei GH, Liu D et al. Whole-genome and Transcriptome Sequencing of Prostate Cancer Identify New Genetic Alterations Driving Disease Progression. Eur Urol 2018; 73: 322-339.

4. Beltran H, Prandi D, Mosquera JM et al. Divergent clonal evolution of castration-resistant neuroendocrine prostate cancer. Nat Med 2016; 22: 298-305.

5. Kumar A, Coleman I, Morrissey C et al. Substantial interindividual and limited intraindividual genomic diversity among tumors from men with metastatic prostate cancer. Nat Med 2016; 22: 369-378.

6. Robinson D, Van Allen EM, Wu YM et al. Integrative clinical genomics of advanced prostate cancer. Cell 2015; 161: 1215-1228.

7. Gao J, Aksoy BA, Dogrusoz U et al. Integrative analysis of complex cancer genomics and clinical profiles using the cBioPortal. Sci Signal 2013; 6: pl1.

8. Cerami E, Gao J, Dogrusoz U et al. The cBio cancer genomics portal: an open platform for exploring multidimensional cancer genomics data. Cancer Discov 2012; 2: 401-404.
